# Sex Differences in Context-Driven Reinstatement of Methamphetamine Seeking is Associated with Distinct Neuroadaptations in the Dentate Gyrus

**DOI:** 10.3390/brainsci8120208

**Published:** 2018-11-28

**Authors:** Yoshio Takashima, Joyee Tseng, McKenzie J. Fannon, Dvijen C. Purohit, Leon W. Quach, Michael J. Terranova, Khush M. Kharidia, Robert J. Oliver, Chitra D. Mandyam

**Affiliations:** 1 Department of Anesthesiology, University of California San Diego, San Diego, CA 92161, USA; yoshi.takashima@gmail.com; 2 VA San Diego Healthcare System, San Diego, CA 92161, USA; j1tseng@ucsd.edu (J.T.); mfannon@vapop.ucsd.edu (M.J.F.); Dpurohit@ucsd.edu (D.C.P.); lwquach@ucsd.edu (L.W.Q.); mterrano@ucsd.edu (M.J.T.); kkharidi@ucsd.edu (K.M.K.); roliver@vapop.ucsd.edu (R.J.O.)

**Keywords:** granule cell neurons, electrophysiology, GluN, CaMKII, choline acetyltransferase

## Abstract

The present study examined differences in operant responses in adult male and female rats during distinct phases of addiction. Males and females demonstrated escalation in methamphetamine (0.05 mg/kg, i.v.) intake with females showing enhanced latency to escalate, and bingeing. Following protracted abstinence, females show reduced responses during extinction, and have greater latency to extinguish compared with males, indicating reduced craving. Females demonstrated lower context-driven reinstatement compared to males, indicating that females have less motivational significance to the context associated with methamphetamine. Whole-cell patch-clamp recordings on dentate gyrus (DG) granule cell neurons (GCNs) were performed in acute brain slices from controls and methamphetamine experienced male and female rats, and neuronal excitability was evaluated from GCNs. Reinstatement of methamphetamine seeking reduced spiking in males, and increased spiking in females compared to controls, demonstrating distinct neuroadaptations in intrinsic excitability of GCNs in males and females. Reduced excitability of GCNs in males was associated with enhanced levels of neural progenitor cells, expression of plasticity-related proteins including CaMKII, and choline acetyltransferase in the DG. Enhanced excitability in females was associated with an increased GluN2A/2B ratio, indicating changes in postsynaptic GluN subunit composition in the DG. Altered intrinsic excitability of GCNs was associated with reduced mossy fiber terminals in the hilus and pyramidal projections, demonstrating compromised neuroplasticity in the DG in both sexes. The alterations in excitability, plasticity-related proteins, and mossy fiber density were correlated with enhanced activation of microglial cells in the hilus, indicating neuroimmune responses in both sexes. Together, the present results indicate sexually dimorphic adaptive biochemical changes in excitatory neurotransmitter systems in the DG and highlight the importance of including sex as a biological variable in exploring neuroplasticity and neuroimmune changes that predict enhanced relapse to methamphetamine-seeking behaviors.

## 1. Introduction

Clinical and preclinical research indicates that female subjects, compared to male subjects, exhibit greater vulnerability toward drug abuse at stages of the addiction process that mark transitions in drug use, including drug initiation, bingeing, and dependence [1,2,3]. Fewer women currently use methamphetamine than men [4], whereas women show heavier methamphetamine use and display more acute dependence than men [5,6]. Preclinical studies have devoted minimal focus on sex differences in methamphetamine addiction using models of extended access methamphetamine self-administration [7,8,9,10]. Based on the few studies, it is evident that both sexes escalate methamphetamine intake during extended access methamphetamine self-administration conditions with enhanced methamphetamine consumption in females relative to males [9,10]. Next, male rodents experience more severe withdrawal effects than females after psychostimulant use [11], suggesting that elevated methamphetamine use in females may be due to their greater sensitivity to rewarding effects of the drug [12,13] and to their resilience to the negative affect associated with withdrawal. 

With respect to relapse to drug seeking, prior research in rats show that while males and females do not differ in responding on the first day of extinction, females are more likely than males to show resistance to extinction when behavior is tested immediately after cessation of self-administration [14,15]. This sex difference in extinction behavior is not evident when rats experience extinction sessions weeks into abstinence [10,14,16], suggesting that the period of abstinence predicts distinct neurobiological alterations in male and female subjects that may relate to the behavioral responses. Furthermore, females show higher reinstatement of drug seeking behavior compared to males when reinstatement is triggered by the drug, stress, or pharmacological stressors [7,10,14,16], however, males show higher seeking when reinstatement is triggered by drug cues [15]. These data suggest that differences in propensity to reinstatement in males and females is dependent on the reinstatement stimulus (i.e., drug, stress, or cue). Given these stimulus dependent sex differences in drug seeking, it is notable that sex differences in relapse to drug seeking driven by drug context have not been investigated. 

Preclinical studies on sex differences in neural function in response to methamphetamine have been recently evaluated. For example, males display greater methamphetamine-induced striatal neurotoxicity than females [17]. However, sexual dimorphisms in neural activity in response to methamphetamine-conditioned cues are unknown, and were investigated in this study. We compared functional plasticity of granule cell neurons (GCNs) in the dentate gyrus (DG) of the hippocampus in concert with biochemical measures of neuronal excitability and inhibition, developmental stages of newly born GCNs, and neuroimmune responses in the DG in male and female rats immediately following context-driven reinstatement to methamphetamine seeking. 

## 2. Materials and Methods

### 2.1. Animals

**Figure 1:**
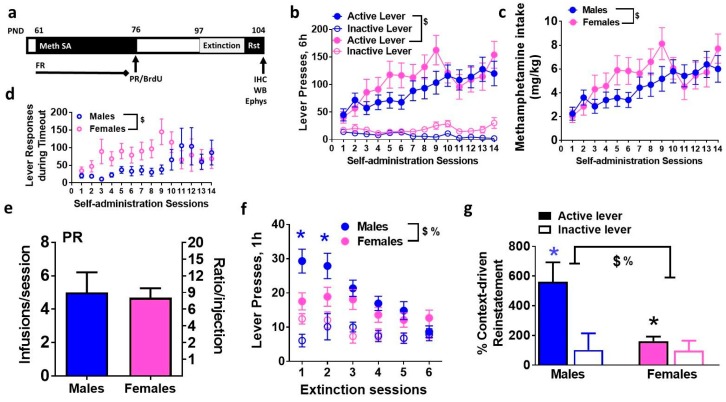
Distinct operant responses during extended access methamphetamine self-administration sessions in male and female rats. (**a**) Schematic of the experimental design indicating the age of animals in days (PND, postnatal days) and sessions during operant behavior. FR, fixed-ratio schedule; PR, progressive-ratio schedule; Rst, reinstatement; IHC, immunohistochemistry; WB, Western blotting; Ephys, electrophysiology. (**b**) Active and inactive lever responses of male and female rats self-administering 0.05 mg/kg/infusion of methamphetamine. (**c**) Total amount of methamphetamine consumed per session. (**d**) Active lever responses during timeout period during each session. (**e**) Lever responses during PR sessions. (**f**) Active and inactive lever responses during extinction sessions. (**g**) Percent context-driven reinstatement, calculated as a percent change in lever responses during reinstatement from last day of extinction. Data are represented as mean ± S.E.M. *n* = 22 males, *n* = 17 females. %, significant sex × methamphetamine interaction; $, main effect of sex; * *p* < 0.05 vs. inactive levers in males; * *p* < 0.05 vs. males.

Experimental procedures were carried out in strict adherence to the NIH Guide for the Care and Use of Laboratory Animals and approved by the Institutional Animal Care and Use Committee of VA San Diego Healthcare System. Thirty-seven adult male and twenty-eight adult female Long Evans rats (bred at the VA Vivarium), age matched, males weighing 200–250 g and females weighing 165–190 g, and 8 weeks old at the start of the experiment (Figure 1a), were housed two per cage in a temperature-controlled vivarium under a reverse light/dark cycle (lights off 8:00 AM–8:00 PM) and completed the study. Self-administration behavior of *n* = 14 male rats and electrophysiology data of *n* = 2 control male rats and *n* = 3 methamphetamine male rats have been previously published elsewhere. Behavior from additional rats (*n* = 8 male rats) and electrophysiology from additional rats (*n* = 2 control rats and *n* = 2 methamphetamine male rats) have been added to the previously published data and are reported here. Behavior and electrophysiology data from female rats have not been published previously. Postmortem tissue analysis of all male and female rats were performed separately for this study and data analysis was done to compare males with females.

### 2.2. Training and Maintenance on an Extended Access Schedule of Intravenous Methamphetamine Self-Administration

Twenty-two male and seventeen female rats underwent surgery for catheter implantation for intravenous self-administration. Rats were implanted with a sterilized silastic catheter into the right jugular vein under aseptic conditions and anesthesia. The distal end of the catheter was threaded under the skin to the back of the rat and exited the skin via a metal guide cannula [18]. Post-surgery care was provided with analgesics (Flunixin) and antibiotics (Cefazolin [19]). Catheters were flushed daily with heparinized saline and tested for patency using methohexital sodium (Brevital [18]). Four to five days after surgery, all rats were trained in an A/B/A between session reinstatement procedure [20]. Animals were initially trained to press a lever according to fixed-ratio 1 (FR1) schedule of methamphetamine reinforcement (0.05 mg/kg/injection of methamphetamine (generously provided by NIDA)) in operant boxes (context A; Med Associates) under extended access conditions (6 h access per day for 14 days). In context A, during daily sessions, a response on the active lever resulted in a 4 s infusion (90–100 µL of methamphetamine), followed by a 20 s timeout period to prevent overdose. Each infusion was paired for 4 s with white stimulus light over the active lever (conditioned stimulus (CS)). Response during the time-out or on the inactive lever was recorded but resulted in no programmed consequences. All animals acquired methamphetamine self-administration and experienced 14 sessions of extended access schedule after the priming sessions.

### 2.3. Progressive Ratio

The following day after extended access sessions, responding was reinforced on a progressive ratio (PR) schedule of reinforcement [21]. On this schedule, the number of responses required for reinforcement incremented progressively, and each session continued until a breakpoint (defined as the number of infusions obtained before 1 h elapsed with no infusions) was reached. For example, in the PR schedule, the response requirement began at 1 response/injection and increased according to the following equation: responses/injection = (5 × e^(injection number × 0.2)^) − 5 [22]. When a rat failed to achieve the response requirement within 1 h, the session ended. A session length under a PR schedule was always set at 6 h, and PR sessions did not exceed 3 h across rats. Breakpoints were determined for 0.05 mg/kg/infusion of methamphetamine.

### 2.4. Extinction

Three weeks after PR session, extinction sessions were performed in a new context (operant box context B, distinct from context A) for 1 h for six sessions. In this context B, animals were not attached to the drug infusion apparatus, white noise was added during the entire session, a house light was turned on for the entire session, and black colored tape was pasted on the operant door. Responses on either the active or inactive lever were recorded and did not result in programmed consequences (i.e., no infusions and no conditioned stimulus presentations). In animals that demonstrated spontaneous seeking (evaluated as significant differences between active and inactive lever responses) on day 1 of extinction, extinction of spontaneous seeking was defined as reduced (<30%) active lever pressing on days 4–6 vs. day 1 of extinction and lack of significant differences between active and inactive lever responses [20].

### 2.5. Reinstatement

Twenty-four hours after the final extinction session, animals underwent context-driven reinstatement in which they were placed into the methamphetamine-paired context (context A) for 1 h, during which they were connected to the infusion apparatus to allow for a similar interaction with the spatial elements of the context as during methamphetamine self-administration training. Lever presses were used as a measure of drug seeking, and responses on either the active or inactive lever were recorded and did not result in an infusion of fluids through the catheter or other programmed consequences (i.e., conditioned stimulus presentations). One hour after the end of the session, animals were either anesthetized and brain tissue was processed for electrophysiology or euthanized by rapid decapitation for Western blotting or immunohistochemistry. 

At the same time, age matched behavior naïve male and female rats were euthanized and were used as controls. We have previously reported that extended access to saline self-administration, followed by extinction and reinstatement does not alter neurogenesis, the number of progenitors in the DG, and plasticity related proteins in the DG compared to age matched behavior naïve controls [23]. We therefore used behavior naïve controls in this study. 

### 2.6. Estrus Cycle Tracking

After the reinstatement session, all female rats were vaginally swabbed with a sterile cotton swab soaked in 0.9% saline. Samples were applied to Superfrost^®^ Plus slides and dried overnight and stained with Cresyl Echt Violet Solution (Abcam, Cambridge, MA, USA) to determine stage of estrus based on cell morphology [24]. 

### 2.7. Slice Preparation and Electrophysiology

In our recently published study [25], electrophysiology data from control males (*n* = 2 rats, *n* = 8 cells) and methamphetamine experienced males (*n* = 3 rats, *n* = 6 cells) have been reported. In this study, additional slices for electrophysiology were prepared from control males (*n* = 2 rats, *n* = 5 cells) and methamphetamine experienced rats (*n* = 2 rats, *n* = 9 cells) and electrophysiology data is combined and reported here. The number of animals and cells for electrophysiology are: methamphetamine naïve (control males, *n* = 4 rats, *n* = 13 cells; control females, *n* = 3 rats, *n* = 6 cells) and methamphetamine experienced rats (males, *n* = 5 rats, *n* = 15 cells; females, *n* = 3 rats, *n* = 19 cells). Rats from each group were subjected to brief Ketamine/Xylazine/Acepromazine anesthesia and perfused for 3 min with ice-cold, oxygenated modified sucrose artificial cerebrospinal fluid (ACSF). Slices of 330 µm-thickness containing regions of interest were cut on a vibratome (Leica VT1200; Leica Biosystems, Buffalo Grove, IL, USA) and incubated at 34 °C in an interface chamber containing the same modified sucrose ACSF solution for 30 min. Following incubation at 34 °C slices were held at room temperature (23 °C) in the same chamber for at least 45 min before initiating recordings. Recordings were made in a submersion-type recording chamber and superfused with oxygenated ACSF at 23 °C at a rate of 2–3 mL/minute. Whole-cell patch clamp recordings were obtained using Multiclamp 700B amplifiers (Molecular Devices, San Jose, CA, USA) and data was collected using pClamp 10 software (Molecular Devices, San Jose, CA, USA). Data was low-pass filtered at 2 kHz, and digitized at 10 kHz (Digidata 1440A; Molecular Devices, San Jose, CA, USA). Voltage clamp recordings were made at room temperature using glass-pulled patch pipettes (Warner Instruments, OD = 1.50 mm, ID = 1.16 mm, length = 10 cm; 4–7 MΩ) filled with internal solution. GCNs in the dorsal DG were visualized and then targeted for whole-cell recording under infrared differential interference contrast videomicroscopy. Recordings were made from GCNs located in the middle or the outer third of the granule cell layer. Cells that were impaled were first screened to ensure that they were healthy. In voltage-clamp experiments the holding membrane potential was set to −68 mV. Series and input resistances were monitored before and after recordings; data were discarded if it increased by >10 MΩ. In current-clamp experiments, currents were injected stepwise, in 20 pA increments. Step duration was 500 ms.

Analysis of electrophysiological properties was made with Clampfit 10.4 software (Molecular Devices, San Jose, CA, USA) as previously described [25]. In brief, resting potential is defined as the recorded membrane potential when cell membrane was initially broken into with a recording pipette for the whole-cell patch clamp configuration. Action potential (AP) characteristics are based on a single AP evoked by intracellular current injection (ranging between 60 to 180 pA, 500 ms pulse). Peak amplitude is actual measured peak AP amplitude in mV. Spike threshold is defined as the voltage at which the second derivative of the membrane potential trajectory approaching a spike crossed a user-selected threshold of 20 mV/ms^2^. Half-width is the period measured in latency (ms) from the start of the AP to the time when the AP reached half of the peak amplitude. The amplitude of afterhyperpolarization (AHP) was measured from the threshold voltage to the most negative overshoot after repolarization. The slope of the rise to threshold was obtained by a linear fit from completion of the AHP to the threshold of the subsequent AP. Interspike intervals (ISIs) were measured as the time between first evoked AP peak to the second AP.

### 2.8. 5-bromo-2′-deoxyuridine (BrdU) Injections

Rats received one intraperitoneal injection of 150 mg/kg BrdU (Boehringer Mannheim Biochemica) dissolved in 0.9% saline and 0.007 N NaOH at 20 mg/mL. Methamphetamine rats (*n* = 4 males, *n* = 7 females) were injected with BrdU after completion of their PR session; control rats (*n* = 4 males, *n* = 4 females) were injected on the same day. 

### 2.9. Brain Tissue Collection for Immunohistochemistry and Western Blotting

BrdU injected rats and rats without BrdU (*n* = 8 control males, *n* = 4 control females, *n* = 15 reinstated males, *n* = 6 reinstated females) were killed by rapid decapitation and the brains were isolated, and dissected along the midsagittal plane. The left hemisphere was snap frozen for Western blotting analysis and the right hemisphere was postfixed in 4% paraformaldehyde for immunohistochemistry [26]. For immunohistochemistry, the tissue was sliced in 40 µm sections along the coronal plane on a freezing microtome. 

### 2.10. Immunohistochemistry (IHC)

Every eighteenth section through the hippocampus (anterio-posterior −2.5 to −6.3 mm from bregma) was mounted on Superfrost^®^ Plus slides and dried overnight [27]. Sections were stained for Ki-67 (1:700, Rabbit polyclonal, Thermo Scientific, Waltham, MA, USA), doublecortin (DCX, 1:500, Goat polyclonal, Santacruz Biotechnology, Dallas, TX, USA), BrdU (1:400; sheep polyclonal, Abcam, Cambridge, MA, USA), synaptoporin (1:50; rabbit polyclonal, Synaptic Systems, Göttingen, Germany), and ionized calcium-binding adapter molecule 1 (Iba-1, 1:1000, Wako, Richmond, VA, USA), followed by biotin-tagged secondary antibodies and visualized with DAB. Immunoreactive cells in the subgranular zone (SGZ; i.e., cells that touched and were within three cell widths inside and outside the hippocampal granule cell-hilus border for Ki-67) and cells in the granule cell layer (BrdU) were quantified with a Zeiss AxioImagerA2 (400× magnification) using the optical fractionator method, in which sections through the DG (−1.4 to −6.7 mm from bregma [28]) were examined. In addition to cell counting, area measures of the granule cell layer was determined for each section for each animal using StereoInvestigator software (MicroBrightField, Williston, VT, USA), and the raw cell counts per section per animal for Ki-67, BrdU were divided by the area of the granule cell layer and are indicated as cells per 2 mm of the granule cell layer per animal. The density of DCX in hippocampal sections through the DG (−1.4 to −6.7 mm from bregma) was measured. The images were captured with Zeiss AxioImagerA2 at 10× to incorporate the entire granule cell layer. Greyscale, white-balanced images were captured with StereoInvestigator software and were evaluated by quantifying DAB stain (% area stained) using ImageJ software (NIH). Briefly, DCX cells were contoured using the polygonal selection feature. A circular area above the granule cell layer was used to quantify non-specific/background staining. 

For morphometric analysis of the density of mossy fiber projections, dorsal hippocampal sections (representing −2.56 and −4.8 mm from bregma, 4 sections per rat) were separately stained for synaptoporin [29]. For mossy fiber density, images were captured at 10× and the mossy fiber tracts were combined for density measures. Colored, white-balanced images were captured with StereoInvestigator software; synaptoporin in the hilus of the dorsal DG and the stratum lucidum (where mossy fibers synapse with hilar and CA3 neurons, (pyramidal projections)) were evaluated by quantifying DAB stain (% area stained) using ImageJ software (NIH). Briefly, the mossy fiber tracts were contoured using the polygonal selection feature. A circular area above the CA3 was used to quantify non-specific/background staining. 

For both the DCX and mossy fiber density, the images was then converted to red-green-blue stacks. The green stack was used for quantification of DAB using the threshold function; the maximum and minimum threshold for all the images was set to 130 and 90, respectively. The area was stained (% area) and the background was measured; specific staining was calculated by subtracting the background [30]. 

### 2.11. Analysis of Microglial Cells

Sections of the hippocampus, −2.56 to −3.8 mm to bregma were stained with rabbit anti-Iba-1 (019-19741, 1:1000; Wako, Richmond, VA, USA) to determine changes in structure of microglial cells. To evaluate Iba1 morphology, a Zeiss Axiophot microscope and a computer-based system (Neurolucida; Micro-BrightField, Williston, VT, USA) was used to generate three-dimensional cell tracings that were subsequently visualized and analyzed using NeuroExplorer (MicroBrightField, Williston, VT, USA). In order for a cell to be selected the following four criteria were met: (1) the cell was in the region of interest (in the hilus touching the outer granule cell layer of the superior or inferior blade), (2) the cell was distinct from other surrounding cells to allow for identification of processes, (3) the cell was not truncated or broken, and (4) the cell exhibited dark, well filled staining [25].

For each animal, 6 cells were traced at 40× magnification with an oil immersion lens (equipped with a 10× eye piece), and morphological measurements were analyzed separately. A 3D Sholl analysis was performed in which concentric spheres of increasing radius (starting sphere 5 µm and increasing in 1 µm increments) were layered around the cell body until processes were completely encompassed. For each reconstructed cell, an estimate of area of the cell body and length of processes was obtained using the Sholl ring method. 

### 2.12. Western Blotting

Animals were euthanized via rapid decapitation under light isoflurane anesthesia (3–5%) 1 h after end of reinstatement session. Brains were quickly removed and flash-frozen. Brain tissue was cut at the mid-sagittal axis and the right hemisphere was processed for Western blotting [31]. Tissue punches from dorsal hippocampus enriched in the DG (did not include CA3 or CA1 areas) from 500 μm thick sections were homogenized on ice by sonication in buffer (320 mM sucrose, 5 mM HEPES, 1 mM EGTA, 1 mM EDTA, 1% SDS, with Protease Inhibitor Cocktail and Phosphatase Inhibitor Cocktails II and III diluted 1:100; Sigma, St. Louis, MO, USA), heated at 100 °C for five minutes, and stored at −80 °C until determination of protein concentration by a detergent-compatible Lowry method (Bio-Rad, Hercules, CA, USA). Samples were mixed (1:1) with a Laemmli sample buffer containing β-mercaptoethanol. Each sample containing protein from one animal was run (20 μg per lane) on 8% SDS-PAGE gels (Bio-Rad, Hercules, CA, USA) and transferred to polyvinylidene fluoride membranes (PVDF pore size 0.2 μm). Blots were blocked with 2.5% bovine serum albumin (for phosphoproteins) or 5% milk (w/v) in TBST (25 mM Tris–HCl (pH 7.4), 150 mM NaCl and 0.1% Tween 20 (v/v)) for 16–20 h at 4 °C and were incubated with the primary antibody for 16–20 h at 4 °C: antibody to total CaMKII (tCaMKII) (1:200; catalog #ab52476, Abcam, Cambridge, MA, USA; molecular weights, 47 and 60 kDa); tGluN2A (1:200, Santa Cruz Biotechnology, Dallas, TX, USA; cat. no. sc-9056, molecular weight 170 kDa); GluN2B (1:200, Santa Cruz, Dallas, TX, USA; cat. no. sc-9057, molecular weight 180 kDa) and choline acetyltransferase (ChAT, 1:200, Millipore, St. Louis, MO, USA; cat. no. AB144P, molecular weight 70 kDa). Blots were then washed with TBST and incubated for 1 h at room temperature with horseradish peroxide-conjugated goat antibody to rabbit or horseradish peroxide-conjugated goat antibody to mouse (1:5000, BioRad, Hercules, CA, USA) in TBST. Following subsequent washes, immunoreactivity was detected using SuperSignal West Dura chemiluminescence detection reagent (Thermo Scientific, Waltham, MA, USA) and images were collected using a digital imaging system (Azure Imager c600, VWR, Radnor, PA, USA). For normalization purposes, membranes were incubated with 0.125% coomassie stain for 5 min and washed three times for 5–10 min in destain solution. Densitometry was performed using ImageJ software (NIH). The signal value of the band of interest following subtraction of the background calculation was then expressed as a ratio of the corresponding coomassie signal (following background subtraction). This ratio of expression for each band was then expressed as a percent of the drug naïve control male animals included on the same blot.

### 2.13. Statistical Analyses

The methamphetamine self-administration data is expressed as the total number of lever responses during the sessions of methamphetamine self-administration. The effect of session duration on methamphetamine self-administration during the 6 h session was examined over the 14 escalation sessions using a repeated-measures analysis of variance (ANOVA) followed by the Student-Newman-Keuls post hoc test. Differences in the rate of responding between the first and other escalation sessions were evaluated using the paired *t*-test. The effect of forced abstinence on extinction and reinstatement was examined using a repeated-measures (ANOVA) followed by the Student-Newman-Keuls post hoc test. For BrdU, Ki-67, DCX, synaptoproin and Iba1 analysis, two-way ANOVA was conducted to determine interactions and group differences. For Western blotting analyses two-way ANOVA was conducted on raw data and percent change from respective controls was used for graphical representation. For electrophysiological studies, repeated measures two-way ANOVA was used to determine differences in intrinsic excitability, two-way ANOVA was used to detect differences in all other neuronal firing characteristics. When there were significant interactions post-hoc analysis were made with Student-Newman-Keuls post hoc test. The data are expressed as mean ± SEM in all graphs.

## 3. Results

### 3.1. Extended Access to Meth Self-Administration Resulted in Escalation of Meth Intake in Male and Female Rats 

Self-administration behavior of *n* = 14 male rats have been previously published elsewhere [25]. Behavior from additional rats (*n* = 8 male rats) have been added to the previously published data and are reported here. Rats experienced methamphetamine self-administration on a fixed-ratio schedule for 14 days (Figure 1a,b). Repeated measures two-way ANOVA of active lever responses did not detect a significant session × sex interaction (Figure 1b, F_13, 526_ = 0.8, *p* = 0.6), however, detected a significant effect of sex (Figure 1b, F_1, 526_ = 6.2, *p* = 0.01) and sessions (Figure 1b, F_13, 526_ = 3.8, *p* < 0.001). Active lever responses during timeout did not change over days of self-administration. Repeated measures two-way ANOVA of timeout responses did not detect a significant session × sex interaction and main effect of sessions, however, detected a significant effect of sex (Figure 1d, F_1, 526_ = 9.2, *p* = 0.002). No significant changes were observed in inactive lever responses. After the last fixed-ratio schedule session, all rats were tested on a PR schedule to identify differences in motivation to obtain methamphetamine. No differences were observed between males and females in the number of methamphetamine infusions that were earned (*p* = 0.53, Figure 1e).

### 3.2. Males and Females Distinctly Extinguish Operant Behavior and Demonstrate Varied Context-Driven Reinstatement of Methamphetamine Seeking

After 21 days of forced abstinence from methamphetamine, rats experienced extinction sessions. During extinction sessions, methamphetamine was not available and lever responding on the first and second day of extinction significantly differed between males and females, as seen in Figure 1f. Repeated measures two-way ANOVA of active lever responses detected a significant sex × session interaction, F_5, 221_ = 2.2, *p* = 0.05; a significant effect of sex, F_1, 221_ = 8.3, *p* = 0.004; and effect of session F_5, 221_ = 8.3, *p* < 0.0001. Post-hoc analysis demonstrated higher responding in males on the first and second day of extinction compared with females (Figure 1f; *p* < 0.05). Males and females extinguished operant responses. Following extinction, rats were tested on context-driven reinstatement (Figure 1g). All male rats showed higher responding on the previously drug-paired lever during context-driven reinstatement when compared with the last extinction session (lever responses on the previously paired active lever: males; extinction day 6 − 7.8 ± 1.5, reinstatement- 22.2 ± 4.2, paired *t* test; *p* = 0.004; females; extinction day 6 − 13.2 ± 3.0, reinstatement- 17.9 ± 3.1, paired *t* test; *p* = 0.24). Two-way ANOVA demonstrated a significant sex × lever interaction (F_1, 63_ = 6.1, *p* = 0.01), main effect of sex F_1, 63_ = 6.4, *p* = 0.01 and main effect of lever F_1, 63_ = 10.8, *p* = 0.001 when percent context-driven reinstatement was analyzed (Figure 1g). Post-hoc analysis revealed higher reinstatement in males compared to females (*p* = 0.001; Figure 1g). We determined the estrus phase of females in control and reinstated rats and the number of females in each phase in methamphetamine rats did not significantly differ from controls and the number of lever responses during reinstatement did not differ between females in each phase. 

### 3.3. Immunohistochemical Analysis with Markers of Proliferation, Differentiation and Survival Reveal Distinct Effects of Methamphetamine and Abstinence on Progenitor Cells in Males and Females

**Figure 2:**
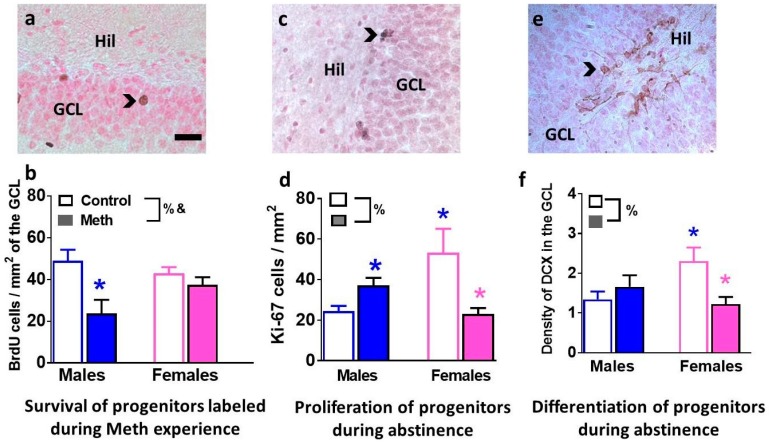
Developmental stages of neurogenesis in the dentate gyrus (DG) are distinctly altered in males and females. (**a**,**c**,**e**) Photomicrographs of BrdU (**a**), Ki-67 (**c**) and doublecortin (DCX) (**e**) from one control male rat. Arrowhead points to immunoreactive cells in each panel. Hil, Hilus; GCL, granule cell layer. Scale bar in (**a**) is 20 µm, it applies to a, c, e. (**b**,**d**,**f**) Quantitative analysis of BrdU (**b**), Ki-67 (**c**) and DCX (**e**). Data are represented as mean ± S.E.M. *n* = 4–19 males, *n* = 4–13 females. %, significant sex × reinstatement interaction; &, main effect of reinstatement; * *p* < 0.05 vs. control males; * *p* < 0.05 vs. control females.

BrdU was injected to label neural progenitor cells (NPCs) that were born when rats had consumed methamphetamine. Twenty-eight-day-old BrdU cells were quantified to determine whether survival of NPCs born during methamphetamine experience was distinctly altered in males and females (Figure 2a). Two-way ANOVA demonstrated a significant sex × reinstatement interaction (F_1, 15_ = 4.2, *p* = 0.05) and main effect of reinstatement F_1, 15_ = 10.3, *p* = 0.005 when numbers of BrdU cells were analyzed (Figure 2b). Post-hoc analysis revealed lower number of BrdU cells in reinstated males compared to control males (*p* < 0.05).

Immunohistochemistry with Ki-67 (marker for actively dividing NPCs) and DCX (marker for immature neurons) was performed to determine the effect of reinstatement on developmental stages of NPCs (Figure 2c,e). Two-way ANOVA demonstrated a significant sex × reinstatement interaction (F_1, 23_ = 12.9, *p* = 0.001) when numbers of Ki-67 cells were analyzed (Figure 2d). Post-hoc analysis revealed lower numbers of Ki-67 cells in females that experienced reinstatement and control males compared to control females, and higher numbers of Ki-67 cells in reinstated males compared to control males (*p* < 0.05; Figure 2d). Two-way ANOVA demonstrated a significant sex × reinstatement interaction (F_1, 46_ = 6.2, *p* = 0.01) when density of DCX cells were analyzed (Figure 2f). Post-hoc analysis revealed lower levels of DCX cells in females that experienced reinstatement and control males compared to control females (*p* < 0.05; Figure 2f).

### 3.4. Intrinsic Firing Properties of GCNs are Differentially Altered in Male and Female Rats that Experienced Context-Driven Methamphetamine Seeking

**Figure 3:**
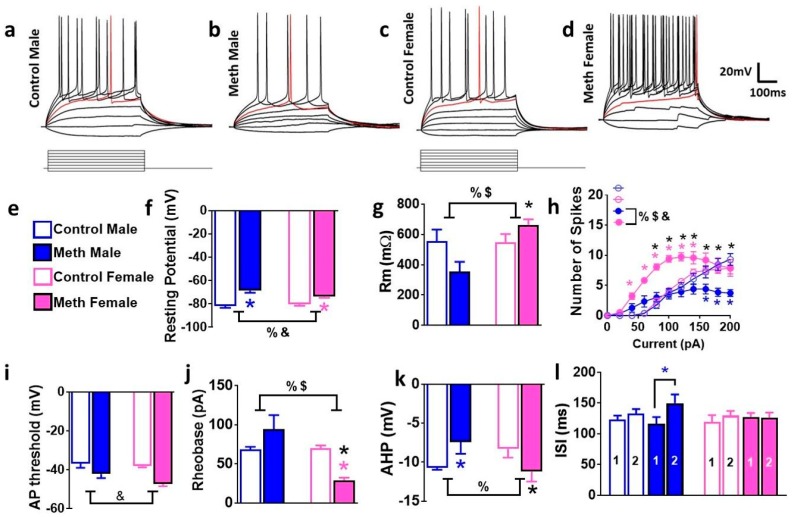
Electrophysiological properties of granule cell neurons (GCNs) are distinctly altered in male and female rats. (**a–d**) Representative traces of action potentials elicited by depolarizing current injections. Traces were recorded from GCNs from control male (**a**), methamphetamine male (**b**), control female (**c**), and methamphetamine female (**d**) rats. (**e**) Legends for bar graphs. (**f–l**) Comparison of intrinsic and active membrane properties. Analysis across all cells indicated that under baseline conditions resting potential (**f**) and membrane resistance (**g**) showed a significant sex × treatment interaction. (**h**) Graphical relationship between the number of spikes elicited by increasing current injections in current-clamp recording. (**i–l**) Analysis of electrophysiological properties indicate that AP threshold, rheobase, AHP, and inter-spike interval (ISI) showed significant interaction or main effect of sex or methamphetamine. Data shown are represented as mean ± SEM. Control males, *n* = 8 cells; control females, *n* = 6 cells; reinstated males, *n* = 12 cells; reinstated females, *n* = 19 cells. %, significant sex × reinstatement interaction; $, main effect of sex; * *p* < 0.05 vs. control males; * *p* < 0.05 vs. reinstated males; * *p* < 0.05 vs. control females by post-hoc tests.

We next studied the intrinsic excitability of GCNs in male and female rats from control and reinstatement groups (Figure 3a–d). GCNs from male and female reinstated rats showed depolarized resting potentials compared with controls (Figure 3f). Two-way ANOVA demonstrated a significant sex × reinstatement interaction (F_1, 39_ = 4.9, *p* = 0.03) and a main effect of reinstatement (F_1, 39_ = 41.8, *p* < 0.0001). Post-hoc analysis revealed more depolarized resting potential in reinstated males and females compared to controls and more depolarized resting potential in reinstated males compared to females that experienced reinstatement (*p* < 0.05; Figure 3f). GCNs from male and female reinstated rats showed distinct alterations in membrane resistance compared with controls (Figure 3g). Two-way ANOVA demonstrated a significant sex × reinstatement interaction (F_1, 39_ = 7.6, *p* = 0.008) and a main effect of sex (F_1, 39_ = 6.7, *p* = 0.01). Post-hoc analysis revealed lower membrane resistance in reinstated males compared to females (*p* < 0.05; Figure 3g).

Depolarizing current injections generated fast action potentials with large amplitudes in GCNs from males and females (Figure 3h). The number of spikes elicited by GCNs from each group with increasing current injections in current-clamp recording were determined, and repeated measures two-way ANOVA demonstrated a significant number of spikes × reinstatement interaction (F_30, 450_ = 4.3, *p* < 0.001), significant increases in the number of spikes over current injections (F_10, 450_ = 59.4, *p* < 0.001) and significant effect of reinstatement (F_3, 45_ = 11.2, *p* < 0.001). Post hoc analysis demonstrates that female rats that experienced reinstatement have increased number of spikes with increasing current injections compared to female controls from current injections ranging from 40 pA to 140 pA, male reinstated rats have reduced number of spikes with increasing current injections compared to male controls from current injections ranging from 160 pA to 200 pA, and female rats that experienced reinstatement have increased number of spikes with increasing current injections compared to male reinstated rats from current injections ranging from 80 pA to 200 pA (Figure 3h; *p*s < 0.05). At higher current injections (>200 pA), there were no significant differences in the number of spikes between controls and reinstated rats, indicating that GCNs from all groups elicited property of regular spiking by depolarizing current injections (data not shown). 

The distinct intrinsic firing properties of GCNs in methamphetamine male and female rats were associated with parallel alterations in AP threshold, rheobase current, interspike interval, and AHP (Figure 3i–l). AP threshold analyzed with two-way ANOVA demonstrated a significant effect of reinstatement (F_1, 39_ = 16.1, *p* = 0.0003). Rheobase current analyzed with two-way ANOVA demonstrated a significant sex × reinstatement interaction (F_1, 39_ = 5.9, *p* = 0.01) and a main effect of sex (F_1, 39_ = 5.2, *p* = 0.02). Post-hoc analysis revealed lower rheobase current in females that experienced reinstatement compared to control females, control males, and reinstated males (*p*s < 0.05; Figure 3j). AHP analyzed with two-way ANOVA demonstrated a significant sex × reinstatement interaction (F_1, 39_ = 10.8, *p* = 0.003). Post-hoc analysis revealed smaller AHP in reinstated males compared to control males and females that experienced reinstatement (*p*s < 0.05; Figure 3k). Repeated measures two-way ANOVA demonstrated a significant difference in inter-spike interval (ISI), measured as spike latency between first and second spike peak (trend towards interaction (F_3, 39_ = 2.5, *p* = 0.08), no effect of treatment and significant effect of ISI (F_1, 39_ = 8.8, *p* = 0.006). Post-hoc analysis showed higher latency to spike between the first and second spike in GCNs from reinstated male rats (*p* < 0.05, Figure 3l).

### 3.5. Expression of Plasticity-Related Proteins in the DG is Differentially Altered by Reinstatement of Methamphetamine Seeking in Males and Females

**Figure 4:**
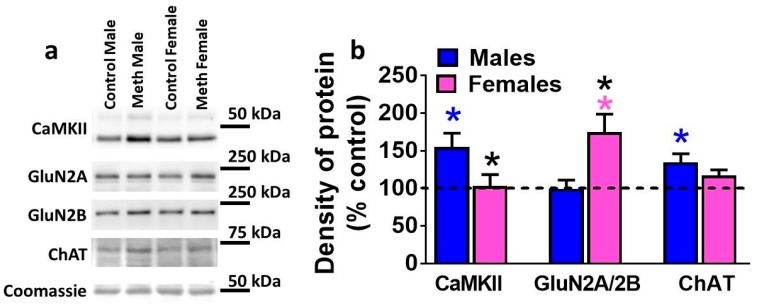
Plasticity related proteins are distinctly affected in male and female rats. (**a**) Representative immunoblots of each protein measured with observed molecular weight in kDa. (**b**) Quantitative analysis of density of proteins shown in b. Data shown are represented as mean ± SEM. *n* = 12–19 males, *n* = 8–13 females. * *p* < 0.05 vs. control males, * *p* < 0.05 vs. reinstated males; * *p* < 0.05 vs. control females by post-hoc tests.

To determine whether reinstatement of methamphetamine seeking altered expression of CaMKII, GluN subunits, and ChAT in the DG, protein levels were analyzed in controls and rats that experienced reinstatement (Figure 4a,b). Data is expressed as percent control, however, statistical significance was calculated from raw density values from control males, reinstated males, control females, and females that experienced reinstatement. Two-way ANOVA demonstrated a significant sex × reinstatement interaction (F_1, 40_ = 5.9, *p* = 0.01) when density of CaMKII was analyzed (Figure 4b). Post-hoc analysis revealed higher expression of CaMKII in reinstated males compared to control males and females that experienced reinstatement (*p* < 0.05; Figure 4b). The ratio of GluN2A to 2B was analyzed in each rat and two-way ANOVA demonstrated a significant sex × reinstatement interaction (F_1, 41_ = 3.9, *p* = 0.05) and significant main effect of reinstatement (F_1, 41_ = 5.4, *p* = 0.02) (Figure 4b). Post-hoc analysis revealed higher ratio of GluN2A/2B in females that experienced reinstatement compared to control females and reinstated males (*p* < 0.05; Figure 4b). Two-way ANOVA demonstrated a significant sex × reinstatement interaction (F_1, 40_ = 4.3, *p* = 0.04) when density of ChAT was analyzed (Figure 4b). Post-hoc analysis revealed higher expression of ChAT in reinstated males compared to control males (*p* < 0.05; Figure 4b).

### 3.6. Density of Mossy Fiber Tracts in the Hilus and Pyramidal Projections of the Dentate Gyrus are Similarly Effected by Context-Driven Methamphetamine in Male and Female Rats

**Figure 5:**
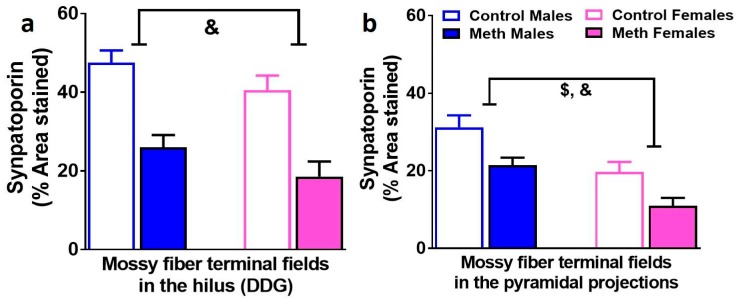
Mossy fiber tracts in the DG are distinctly altered in males and females. (**a**,**b**) Quantitative analysis of the density measures in the hilus (**a**) and CA3 pyramidal projections (**b**). Data shown are represented as the mean ± SEM. *n* = 12–19 males, *n* = 8–13 females. &, main effect of reinstatement; $, main effect of sex by two-way ANOVA.

Immunohistochemistry with synaptoporin was performed to determine whether context-driven methamphetamine differentially effected mossy fiber density in male and female rats (Figure 5a,b). In the hilus, two-way ANOVA demonstrated a significant effect of reinstatement on the density of synaptoporin F_1, 42_ = 25.9, *p* < 0.0002). In the CA3 projections, two-way ANOVA demonstrated a significant effect of reinstatement (F_1, 42_ = 12.2, *p* = 0.001) and sex (F_1, 42_ = 17.9, *p* = 0.0002) on the density of synaptoporin.

### 3.7. Analysis of 3D Structure of Iba-1 Cells Demonstrates Neuroimmune Response in Male and Female Methamphetamine Rats

**Figure 6:**
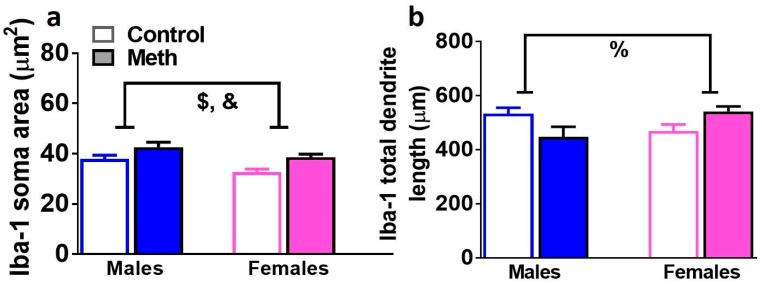
Microglial cells are distinctly altered in male and female rats. 3D Sholl analysis of Iba-1 cells were performed to determine the soma area and total dendrite length of each cell. (**a**,**b**) Quantitative analysis of cell soma area and length of processes of Iba-1 cells. *n* = 6–12 males, *n* = 6–13 females. Data shown are represented as the mean ± SEM. %, sex × reinstatement interaction; &, main effect of reinstatement; $, main effect of sex by two-way ANOVA.

To confirm microglial activation, 3D Sholl analysis of Iba-1 positive cells was performed in controls and male and female rats that experienced reinstatement (Figure 6a,b). When the soma area of Iba-1 cells was analyzed, two-way ANOVA demonstrated a significant effect of reinstatement (F_1, 33_ = 5.2, *p* = 0.02) and sex (F_1, 33_ = 3.9, *p* = 0.05). When the total length of dendrites of Iba-1 cells was analyzed, two-way ANOVA demonstrated a significant sex × reinstatement interaction (F_1, 33_ = 4.6, *p* = 0.03). Post-hoc analysis did not detect any group differences in total length of dendrites.

## 4. Discussion

Previous studies have examined sex differences in animal models of methamphetamine addiction and have focused on differences during self-administration, extinction, and drug- or cue-induced reinstatement of methamphetamine seeking [7,9,10,32,33,34,35,36]. The current study examined whether sex differences occur during context-driven reinstatement of extinguished methamphetamine-seeking behavior following prolonged abstinence from methamphetamine self-administration. Our results support previous findings [9,10] to show that male and female rats differ in their intake of methamphetamine during extended access self-administration sessions, with females taking more drug than males. Studies in rats show that males experience more severe withdrawal effects than females after chronic cocaine intake [11], suggesting that elevated drug use in females may be due to their greater sensitivity to rewarding effects of the drug and to their resilience to the negative affect associated with withdrawal. Alternatively, higher active lever responses in females may be due to increased sensitivity to methamphetamine-induced psychomotor effects, since females exhibit greater locomotion and more stereotypy in response to methamphetamine than males [12,37,38]. This possibility is diminished because inactive lever responses did not differ between males and females, suggesting that active lever responses in females is not a result of hyperactivity. It is also unlikely that the greater active lever responding in females than in males was due to sex differences in body weight, since methamphetamine dosing were proportional to body weight. In contrast, females did not show higher lever responses during PR session, indicating that males and females are equally sensitive to the motivational effects of methamphetamine. This effect is different from a report that showed that females exhibit higher breaking points on a PR schedule of methamphetamine reinforcement, and could be associated with the dose of methamphetamine self-administered and the strain of rats used in the study [9]. 

With respect to relapse to methamphetamine seeking, prior research in rats shows that females and males do not show differences in responding on the first day of extinction when behavior is tested immediately or days after cessation of self-administration [7,10,14,15,32,39]. Our findings reveal sex differences in responding on the first day of extinction, with females showing lower responses or lower spontaneous seeking compared to males when tested weeks into abstinence, indicating that male rats are more susceptible to seeking during extinction (nonreinforced operant performance in the absence of context and cue presentation) and females have reduced motivation to seek during extinction [40]. While the current findings do not provide a time course of extinction sessions during early and protracted periods of abstinence, we speculate that these behavioral differences in males and females could be associated with the period of abstinence. Females show higher reinstatement of drug seeking behavior compared to males when reinstatement is triggered by drug, stress, or pharmacological stressors [7,10,14,16], or when behavior in not extinguished prior to reinstatement [35], however, males show higher seeking when reinstatement is triggered by drug cues [15]. These data suggest that the direction of sex differences during reinstatement is dependent on the reinstatement stimulus (i.e., drug, stress, or cue). Given these stimulus dependent sex differences in drug seeking, it is notable that, sex differences in relapse to drug seeking driven by drug context have not been investigated previously, and were the focus of the current study. Our findings show that males are more susceptible to responding on a methamphetamine-paired lever in the presence of previously methamphetamine-paired context. Collectively, these results provide new findings to show that males may be somewhat more sensitive to the motivational effects of methamphetamine as they showed greater resistance to extinction and greater context-driven reinstatement when methamphetamine reinforcement was discontinued.

The hippocampus plays a substantial role in context-driven reinstatement of methamphetamine seeking [41], and the plasticity in the DG of the hippocampus also regulates context-driven methamphetamine seeking [23,42]. Previously, in male rats we injected BrdU immediately after an extended access methamphetamine self-administration session to tag dividing neuroblasts and progenitor cells during methamphetamine experience and continued extended access sessions for four weeks. Immunohistochemical analysis revealed that extended access methamphetamine self-administration reduced the number of twenty-eight-day-old BrdU cells (mature cells that survived to become GCNs) during methamphetamine experience [43]. Parallel studies were conducted to determine the cellular mechanism underlying reduced survival of BrdU cells, and we showed that extended access methamphetamine self-administration reduced the number of actively dividing preneuronal neuroblasts and increased the number of dividing preneuronal progenitor cells [44]. These mechanistic studies suggest that a decrease in the survival of BrdU cells during methamphetamine self-administration is attributable to the decrease in the ability of neuroblasts to divide and produce stable progenitor cells. 

In contrast to the diminishing effects of methamphetamine on progenitor cells during drug use, withdrawal and abstinence from extended access methamphetamine self-administration produce a rebound effect on progenitor cells. For example, we used BrdU to tag cells either 20 h or two weeks after extended access methamphetamine self-administration, and our findings indicate that withdrawal and abstinence from methamphetamine increase survival of BrdU cells [23,45]. Investigation of endogenous markers of proliferation, e.g., Ki-67-labeled dividing cells (Ki-67 is expressed in actively dividing neural stem cells and provides an estimate of net proliferation [46]) during protracted abstinence indicated that abstinence from drug use increased the population of progenitor cells that were neuroblasts or preneuronal progenitor cells [19,42,45]. While these studies indicate opposing effects of methamphetamine and abstinence on survival of BrdU cells and progenitor pool of cells, it is not known if tagging BrdU cells during methamphetamine experience and following it with abstinence from methamphetamine reduces or increases survival of BrdU cells. This will determine whether neuroblasts and progenitor cells born during methamphetamine experience and born during withdrawal have distinct survival profiles in the DG. Our current study investigated this question, and our findings indicate that survival of BrdU cells born during methamphetamine experience is reduced, and this reduction is not dependent on continued methamphetamine access. Notably, this effect is evident in males that demonstrated context-driven reinstatement of methamphetamine seeking, and is not evident in females that did not show reinstatement of methamphetamine seeking. Our study also investigated whether the experience of reinstatement similarly effected Ki-67 cells in males and females, and our results in males confirmed our previous reports to show that reinstatement increases the number of Ki-67 cells in males compared to controls and, in contrast, our new findings show that the number of Ki-67 cells is reduced compared to controls in females that did not reinstate. We also investigated another endogenous marker of cell maturation, e.g., doublecortin (DCX)-labeled immature neurons (DCX is expressed in cells committed to a neuronal phenotype [47]). Our findings add to our previous reports in males, to show that proliferation and maturation of progenitors is enhanced with reinstatement [42]. Females continue to show reduction in maturation of progenitors in concert with decreases in proliferation of progenitors, indicating that contrasting adaptations in newly born GCNs in the DG may assist with predicting divergent responses during context-driven reinstatement [42]. While the limitation of the current study is that the control groups were behavior naïve, and a potential limitation in the interpretation of these results is that it may be difficult to distinguish the effects of reinstatement of methamphetamine seeking compared with seeking to a non-rewarding substance (e.g., saline), our previous published report demonstrates that extended access saline self-administration did not alter neurogenesis in the DG [23]. We are therefore confident with the current comparison groups and highlight the sex differences in the number of progenitors and neurogenesis in drug naïve state and in animals that experienced reinstatement of methamphetamine seeking. 

In the DG, mature progenitors and newly born GCNs contribute to the density of mossy fiber tracts [48]. We determined the density of mossy fiber projections in males and females that were behavior naïve and that experienced context-driven reinstatement to investigate whether alterations in mature progenitors were associated with alterations in mossy fiber density. Our findings demonstrate that in the hilus and CA3, mossy fiber density was significantly reduced in males and females that experienced reinstatement compared with controls, and therefore, this reduction was independent of context-driven reinstatement as females did not reinstate compared to males. In females, the direction of change in the density of mossy fibers between controls and reinstated animals was similar to DCX; however, in males this relationship was divergent. These findings suggest that changes in the density of axons of mature GCNs are perhaps influenced to a greater extent by mature progenitors in females than males, and in males are compensatory to the effects on mature progenitors. It is also evident that newly born progenitors contribute to neuroimmune responses in the DG [49,50,51,52]. Furthermore, high doses of methamphetamine experience is associated with microglial activation in the DG [53]. Although the cellular structure of microglial cells in the females that experienced reinstatement may support microglial activation, our findings do not support the contribution of microglial activation in the DG in male and females to context-driven reinstatement.

We wanted to determine whether adaptations in the DG during abstinence in males and females were confined to newly born GCNs or were also evident in mature GCNs. We have recently demonstrated that context-driven reinstatement of methamphetamine seeking in males is associated with decreases in excitability of mature GCNs [25]. We add to these findings to show that reduced or lack of context-driven reinstatement of methamphetamine seeking in females is associated with increases in excitability of mature GCNs. Investigating membrane properties of GCNs indicated that GCNs from males and females that experienced reinstatement showed depolarized resting potentials compared with controls, and that the resting potentials are closer to firing thresholds. In females that did not reinstate, neuroadaptive changes in the GCNs indicate that the change in resting membrane potential closer to firing threshold, in concert with increases in membrane resistance, functions to enhance the propagation of depolarizing current and therefore intrinsic excitability. Enhanced intrinsic excitability of GCNs may contribute to synaptic plasticity such as long-term potentiation in the DG [54,55,56,57], which could result in an activity-dependent strengthening of synapses or network stability that is thought to underlie certain types of learning and memory, in our case preservation of extinguished responses. In males that reinstated, the opposing regulation of resting membrane potential and AHP to reduced excitability suggests that distinct neuroadaptations are perhaps compensatory to the others. Regardless, this likely has important implications for the modulation of hippocampal circuitry underlying context-driven reinstatement [56,58].

Increases and decreases in intrinsic excitability of hippocampal neurons may serve as a metaplastic mechanism for memory formation and retention [56,58], suggesting that functionally adaptive modulation of excitability during hippocampal learning could promote network stability. Notably, CaMKII and GluNs are implicated in regulating intrinsic excitability of neurons in the hippocampus and elsewhere [55,56,57,58,59,60,61], suggesting that altered expression of these proteins in the DG could correlate with changes in the neuronal excitability of GCNs. We examined the density of CaMKII and the ratio of GluN2A to GluN2B to show that higher CaMKII levels in the DG in males that reinstated were associated with reduced intrinsic excitability of GCNs, and enhanced GluN2A/2B ratio in the DG in females that did not reinstate were associated with increased excitability of GCNs. These findings support the recent studies that have indicated causation between CaMKII and GluNs in oppositely regulating excitability of hippocampal neurons and their roles in learning and memory dependent on the hippocampus [56,57,58,62].

The neuronal properties of mature GCNs from males indicate that these neurons have dysregulated neuronal functioning in the basal state and altered functional plasticity that were associated with context-driven methamphetamine seeking. It is possible that newly born GCNs during abstinence could affect synaptic transmission in the DG and contribute to the altered intrinsic properties of mature GCNs [42,63,64,65,66,67,68]. For example, newly born GCNs receive inputs from septal-hippocampal neurons, transient innervation from mature GCNs, as well as direct feedback from CA3 pyramidal neurons [69]. Newly born GCNs also receive direct robust input from septal cholinergic neurons, which may be important during the maturation process [69]. It is possible that newly born GCNs receive septal cholinergic innervation at earlier developmental stages [70], as neurotoxic cholinergic forebrain lesions decrease cell proliferation and neurogenesis in the DG [71,72], whereas activation of the cholinergic system with donezepil increases new cell survival [73]. In the context of forebrain inputs to the hippocampus, cholinergic neurons from the medial septum have been demonstrated to be involved in the processes that underlie cocaine reward and addiction [74,75,76], such that cocaine injections and self-administration increased release and turnover of acetylcholine in the hippocampus [74,77]. We therefore determined the release of acetylcholine via measuring the density of ChAT in the DG of male and female rats that experienced reinstatement, as release of acetylcholine positively correlates with expression of ChAT [78]. Our findings show a significant increase in the density of ChAT in males that showed context-driven reinstatement. Future studies are required to determine whether the forebrain-hippocampal circuitry is distinctly altered in males that reinstate drug seeking and whether neurogenesis mechanisms contribute to the metaplastic mechanisms driving context-driven reinstatement.

In conclusion, both males and females demonstrate escalation of methamphetamine intake in an extended-access schedule of reinforcement. Following protracted abstinence, males show greater reinstatement of methamphetamine-seeking when triggered by drug context. Given the significant sex differences in relapse to methamphetamine seeking, and the associated differences in neurogenesis and functional plasticity of GCNs, successful characterization of GCNs in the DG will provide mechanistic understanding of the biology of GCNs in the DG in addiction-like behaviors.

## References

[1] Carroll M.E., Anker J.J., Perry J.L. (2009). Modeling risk factors for nicotine and other drug abuse in the preclinical laboratory. Drug Alcohol. Depend..

[2] Chen K., Kandel D. (2002). Relationship between extent of cocaine use and dependence among adolescents and adults in the United States. Drug Alcohol. Depend..

[3] Brady K.T., Randall C.L. (1999). Gender differences in substance use disorders. Psychiatr. Clin. N. Am..

[4] Hedden S.L. (2015). Behavioral Health Trends in the United States: Results from the 2014 National Survey on Drug Use and Health.

[5] Dluzen D.E., Liu B. (2008). Gender differences in methamphetamine use and responses: A review. Gend. Med..

[6] Rungnirundorn T., Verachai V., Gelernter J., Malison R.T., Kalayasiri R. (2017). Sex Differences in Methamphetamine Use and Dependence in a Thai Treatment Center. J. Addict. Med..

[7] Holtz N.A., Lozama A., Prisinzano T.E., Carroll M.E. (2012). Reinstatement of methamphetamine seeking in male and female rats treated with modafinil and allopregnanolone. Drug Alcohol. Depend..

[8] Kucerova J., Vrskova D., Sulcova A. (2009). Impact of repeated methamphetamine pretreatment on intravenous self-administration of the drug in males and estrogenized or non-estrogenized ovariectomized female rats. Neuro Endocrinol. Lett..

[9] Roth M.E., Carroll M.E. (2004). Sex differences in the acquisition of IV methamphetamine self-administration and subsequent maintenance under a progressive ratio schedule in rats. Psychopharmacology.

[10] Reichel C.M., Chan C.H., Ghee S.M., See R.E. (2012). Sex differences in escalation of methamphetamine self-administration: Cognitive and motivational consequences in rats. Psychopharmacology.

[11] Perry J.L., Nelson S.E., Carroll M.E. (2008). Impulsive choice as a predictor of acquisition of IV cocaine self-administration and reinstatement of cocaine-seeking behavior in male and female rats. Exp. Clin. Psychopharmacol..

[12] Milesi-Halle A., McMillan D.E., Laurenzana E.M., Byrnes-Blake K.A., Owens S.M. (2007). Sex differences in (+)-amphetamine- and (+)-methamphetamine-induced behavioral response in male and female Sprague-Dawley rats. Pharmacol. Biochem. Behav..

[13] Schindler C.W., Bross J.G., Thorndike E.B. (2002). Gender differences in the behavioral effects of methamphetamine. Eur. J. Pharmacol..

[14] Kerstetter K.A., Aguilar V.R., Parrish A.B., Kippin T.E. (2008). Protracted time-dependent increases in cocaine-seeking behavior during cocaine withdrawal in female relative to male rats. Psychopharmacology.

[15] Fuchs R.A., Evans K.A., Mehta R.H., Case J.M., See R.E. (2005). Influence of sex and estrous cyclicity on conditioned cue-induced reinstatement of cocaine-seeking behavior in rats. Psychopharmacology.

[16] Anker J.J., Perry J.L., Gliddon L.A., Carroll M.E. (2009). Impulsivity predicts the escalation of cocaine self-administration in rats. Pharmacol. Biochem. Behav..

[17] Bourque M., Liu B., Dluzen D.E., Di Paolo T. (2011). Sex differences in methamphetamine toxicity in mice: Effect on brain dopamine signaling pathways. Psychoneuroendocrinology.

[18] Galinato M.H., Orio L., Mandyam C.D. (2015). Methamphetamine differentially affects BDNF and cell death factors in anatomically defined regions of the hippocampus. Neuroscience.

[19] Sobieraj J.C., Kim A., Fannon M.J., Mandyam C.D. (2016). Chronic wheel running-induced reduction of extinction and reinstatement of methamphetamine seeking in methamphetamine dependent rats is associated with reduced number of periaqueductal gray dopamine neurons. Brain Struct. Funct..

[20] Shaham Y., Shalev U., Lu L., De Wit H., Stewart J. (2003). The reinstatement model of drug relapse: History, methodology and major findings. Psychopharmacology.

[21] Richardson N.R., Roberts D.C. (1996). Progressive ratio schedules in drug self-administration studies in rats: A method to evaluate reinforcing efficacy. J. Neurosci. Methods.

[22] Wee S., Wang Z., Woolverton W.L., Pulvirenti L., Koob G.F. (2007). Effect of aripiprazole, a partial dopamine D2 receptor agonist, on increased rate of methamphetamine self-administration in rats with prolonged session duration. Neuropsychopharmacology.

[23] Galinato M.H., Lockner J.W., Fannon-Pavlich M.J., Sobieraj J.C., Staples M.C., Somkuwar S.S., Ghofranian A., Chaing S., Navarro A.I., Joea A. (2018). A synthetic small-molecule Isoxazole-9 protects against methamphetamine relapse. Mol. Psychiatry.

[24] Cora M.C., Kooistra L., Travlos G. (2015). Vaginal Cytology of the Laboratory Rat and Mouse: Review and Criteria for the Staging of the Estrous Cycle Using Stained Vaginal Smears. Toxicol. Pathol..

[25] Takashima Y., Fannon M.J., Galinato M.H., Steiner N.L., An M., Zemljic-Harpf A.E., Somkuwar S.S., Head B.P., Mandyam C.D. (2018). Neuroadaptations in the dentate gyrus following contextual cued reinstatement of methamphetamine seeking. Brain Struct. Funct..

[26] Somkuwar S.S., Fannon-Pavlich M.J., Ghofranian A., Quigley J.A., Dutta R.R., Galinato M.H., Mandyam C.D. (2016). Wheel running reduces ethanol seeking by increasing neuronal activation and reducing oligodendroglial/neuroinflammatory factors in the medial prefrontal cortex. Brain Behav. Immun..

[27] Somkuwar S.S., Fannon M.J., Staples M.C., Zamora-Martinez E.R., Navarro A.I., Kim A., Quigley J.A., Edwards S., Mandyam C.D. (2016). Alcohol dependence-induced regulation of the proliferation and survival of adult brain progenitors is associated with altered BDNF-TrkB signaling. Brain Struct. Funct..

[28] Paxinos G., Watson C. (1997). The Rat Brain in Stereotaxic Coordinates.

[29] Staples M.C., Fannon M.J., Mysore K.K., Dutta R.R., Ongjoco A.T., Quach L.W., Kharidia K.M., Somkuwar S.S., Mandyam C.D. (2017). Dietary restriction reduces hippocampal neurogenesis and granule cell neuron density without affecting the density of mossy fibers. Brain Res..

[30] Jensen E.C. (2013). Quantitative analysis of histological staining and fluorescence using Image. J. Anat. Rec..

[31] Kim A., Zamora-Martinez E.R., Edwards S., Mandyam C.D. (2015). Structural reorganization of pyramidal neurons in the medial prefrontal cortex of alcohol dependent rats is associated with altered glial plasticity. Brain Struct. Funct..

[32] Bernheim A., Leong K.C., Berini C., Reichel C.M. (2017). Antagonism of mGlu2/3 receptors in the nucleus accumbens prevents oxytocin from reducing cued methamphetamine seeking in male and female rats. Pharmacol. Biochem. Behav..

[33] Cox B.M., Bentzley B.S., Regen-Tuero H., See R.E., Reichel C.M., Aston-Jones G. (2017). Oxytocin Acts in Nucleus Accumbens to Attenuate Methamphetamine Seeking and Demand. Biol. Psychiatry.

[34] Pittenger S.T., Chou S., Barrett S.T., Catalano I., Lydiatt M., Bevins R.A. (2017). Nicotine- and cocaine-triggered methamphetamine reinstatement in female and male Sprague-Dawley rats. Pharmacol. Biochem. Behav..

[35] Ruda-Kucerova J., Amchova P., Babinska Z., Dusek L., Micale V., Sulcova A. (2015). Sex Differences in the Reinstatement of Methamphetamine Seeking after Forced Abstinence in Sprague-Dawley Rats. Front. Psychiatry.

[36] Weber R.A., Logan C.N., Leong K.C., Peris J., Knackstedt L., Reichel C.M. (2018). Regionally Specific Effects of Oxytocin on Reinstatement of Cocaine Seeking in Male and Female Rats. Int. J. Neuropsychopharmacol..

[37] Becker J.B., Molenda H., Hummer D.L. (2001). Gender differences in the behavioral responses to cocaine and amphetamine. Implications for mechanisms mediating gender differences in drug abuse. Ann. N. Y. Acad. Sci..

[38] Ohia-Nwoko O., Haile C.N., Kosten T.A. (2017). Sex differences in the acute locomotor response to methamphetamine in BALB/c mice. Behav. Brain Res..

[39] Cox B.M., Young A.B., See R.E., Reichel C.M. (2013). Sex differences in methamphetamine seeking in rats: Impact of oxytocin. Psychoneuroendocrinology.

[40] Larson E.B., Wissman A.M., Loriaux A.L., Kourrich S., Self D.W. (2015). Optogenetic stimulation of accumbens shell or shell projections to lateral hypothalamus produce differential effects on the motivation for cocaine. J. Neurosci..

[41] Hiranita T., Nawata Y., Sakimura K., Yamamoto T. (2008). Methamphetamine-seeking behavior is due to inhibition of nicotinic cholinergic transmission by activation of cannabinoid CB1 receptors. Neuropharmacology.

[42] Galinato M.H., Takashima Y., Fannon M.J., Quach L.W., Morales Silva R.J., Mysore K.K., Terranova M.J., Dutta R.R., Ostrom R.W., Somkuwar S.S. (2018). Neurogenesis during Abstinence Is Necessary for Context-Driven Methamphetamine-Related Memory. J. Neurosci..

[43] Mandyam C.D., Wee S., Crawford E.F., Eisch A.J., Richardson H.N., Koob G.F. (2008). Varied access to intravenous methamphetamine self-administration differentially alters adult hippocampal neurogenesis. Biol. Psychiatry.

[44] Yuan C.J., Quiocho J.M., Kim A., Wee S., Mandyam C.D. (2011). Extended access methamphetamine decreases immature neurons in the hippocampus which results from loss and altered development of neural progenitors without altered dynamics of the S-phase of the cell cycle. Pharmacol. Biochem. Behav..

[45] Recinto P., Samant A.R., Chavez G., Kim A., Yuan C.J., Soleiman M., Grant Y., Edwards S., Wee S., Koob G.F. (2012). Levels of neural progenitors in the hippocampus predict memory impairment and relapse to drug seeking as a function of excessive methamphetamine self-administration. Neuropsychopharmacology.

[46] Gerdes J., Lemke H., Baisch H., Wacker H.H., Schwab U., Stein H. (1984). Cell cycle analysis of a cell proliferation-associated human nuclear antigen defined by the monoclonal antibody Ki-67. J. Immunol..

[47] Brown J.P., Couillard-Despres S., Cooper-Kuhn C.M., Winkler J., Aigner L., Kuhn H.G. (2003). Transient expression of doublecortin during adult neurogenesis. J. Comp. Neurol..

[48] Romer B., Krebs J., Overall R.W., Fabel K., Babu H., Overstreet-Wadiche L., Brandt M.D., Williams R.W., Jessberger S., Kempermann G. (2011). Adult hippocampal neurogenesis and plasticity in the infrapyramidal bundle of the mossy fiber projection: I. Co-regulation by activity. Front. Neurosci..

[49] Goodfellow M.J., Shin Y.J., Lindquist D.H. (2018). Mitigation of postnatal ethanol-induced neuroinflammation ameliorates trace fear memory deficits in juvenile rats. Behav. Brain Res..

[50] Klaus F., Paterna J.C., Marzorati E., Sigrist H., Gotze L., Schwendener S., Bergamini G., Jehli E., Azzinnari D., Fuertig R. (2016). Differential effects of peripheral and brain tumor necrosis factor on inflammation, sickness, emotional behavior and memory in mice. Brain Behav. Immun..

[51] Marshall S.A., Geil C.R., Nixon K. (2016). Prior Binge Ethanol Exposure Potentiates the Microglial Response in a Model of Alcohol-Induced Neurodegeneration. Brain Sci..

[52] Peng H., Geil Nickell C.R., Chen K.Y., McClain J.A., Nixon K. (2017). Increased expression of M1 and M2 phenotypic markers in isolated microglia after four-day binge alcohol exposure in male rats. Alcohol.

[53] Buchanan J.B., Sparkman N.L., Johnson R.W. (2010). A neurotoxic regimen of methamphetamine exacerbates the febrile and neuroinflammatory response to a subsequent peripheral immune stimulus. J. Neuroinflamm..

[54] Williams M.R., DeSpenza T., Li M., Gulledge A.T., Luikart B.W. (2015). Hyperactivity of newborn Pten knock-out neurons results from increased excitatory synaptic drive. J. Neurosci..

[55] Zhang M., Hung F.S., Zhu Y., Xie Z., Wang J.H. (2004). Calcium signal-dependent plasticity of neuronal excitability developed postnatally. J. Neurobiol..

[56] Crestani A.P., Krueger J.N., Barragan E.V., Nakazawa Y., Nemes S.E., Quillfeldt J.A., Gray J.A., Wiltgen B.J. (2018). Metaplasticity contributes to memory formation in the hippocampus. Neuropsychopharmacology.

[57] Sametsky E.A., Disterhoft J.F., Ohno M. (2009). Autophosphorylation of alphaCaMKII downregulates excitability of CA1 pyramidal neurons following synaptic stimulation. Neurobiol. Learn. Mem..

[58] Milshtein-Parush H., Frere S., Regev L., Lahav C., Benbenishty A., Ben-Eliyahu S., Goshen I., Slutsky I. (2017). Sensory Deprivation Triggers Synaptic and Intrinsic Plasticity in the Hippocampus. Cereb. Cortex.

[59] Klug J.R., Mathur B.N., Kash T.L., Wang H.D., Matthews R.T., Robison A.J., Anderson M.E., Deutch A.Y., Lovinger D.M., Colbran R.J. (2012). Genetic inhibition of CaMKII in dorsal striatal medium spiny neurons reduces functional excitatory synapses and enhances intrinsic excitability. PLoS ONE.

[60] Nelson A.B., Gittis A.H., du Lac S. (2005). Decreases in CaMKII activity trigger persistent potentiation of intrinsic excitability in spontaneously firing vestibular nucleus neurons. Neuron.

[61] Van Welie I., du Lac S. (2011). Bidirectional control of BK channel open probability by CAMKII and PKC in medial vestibular nucleus neurons. J. Neurophysiol..

[62] Ohno M., Sametsky E.A., Silva A.J., Disterhoft J.F. (2006). Differential effects of alphaCaMKII mutation on hippocampal learning and changes in intrinsic neuronal excitability. Eur. J. Neurosci..

[63] Schmidt-Hieber C., Jonas P., Bischofberger J. (2004). Enhanced synaptic plasticity in newly generated granule cells of the adult hippocampus. Nature.

[64] Brunner J., Neubrandt M., Van-Weert S., Andrasi T., Kleine Borgmann F.B., Jessberger S., Szabadics J. (2014). Adult-born granule cells mature through two functionally distinct states. eLife.

[65] Ge S., Yang C.H., Hsu K.S., Ming G.L., Song H. (2007). A critical period for enhanced synaptic plasticity in newly generated neurons of the adult brain. Neuron.

[66] Marin-Burgin A., Mongiat L.A., Pardi M.B., Schinder A.F. (2012). Unique processing during a period of high excitation/inhibition balance in adult-born neurons. Science.

[67] Dieni C.V., Nietz A.K., Panichi R., Wadiche J.I., Overstreet-Wadiche L. (2013). Distinct determinants of sparse activation during granule cell maturation. J. Neurosci..

[68] Dieni C.V., Panichi R., Aimone J.B., Kuo C.T., Wadiche J.I., Overstreet-Wadiche L. (2016). Low excitatory innervation balances high intrinsic excitability of immature dentate neurons. Nat. Commun..

[69] Vivar C., Potter M.C., Choi J., Lee J.Y., Stringer T.P., Callaway E.M., Gage F.H., Suh H., van Praag H. (2012). Monosynaptic inputs to new neurons in the dentate gyrus. Nat. Commun..

[70] Ide Y., Fujiyama F., Okamoto-Furuta K., Tamamaki N., Kaneko T., Hisatsune T. (2008). Rapid integration of young newborn dentate gyrus granule cells in the adult hippocampal circuitry. Eur. J. Neurosci..

[71] Cooper-Kuhn C.M., Winkler J., Kuhn H.G. (2004). Decreased neurogenesis after cholinergic forebrain lesion in the adult rat. J. Neurosci. Res..

[72] Mohapel P., Leanza G., Kokaia M., Lindvall O. (2005). Forebrain acetylcholine regulates adult hippocampal neurogenesis and learning. Neurobiol. Aging.

[73] Kaneko N., Okano H., Sawamoto K. (2006). Role of the cholinergic system in regulating survival of newborn neurons in the adult mouse dentate gyrus and olfactory bulb. Genes Cells.

[74] Smith J.E., Vaughn T.C., Co C. (2004). Acetylcholine turnover rates in rat brain regions during cocaine self-administration. J. Neurochem..

[75] Franklin T.R., Druhan J.P. (2000). Expression of Fos-related antigens in the nucleus accumbens and associated regions following exposure to a cocaine-paired environment. Eur. J. Neurosci..

[76] Gong W., Neill D.B., Justice J.B. (1995). Increased sensitivity to cocaine place-preference conditioning by septal lesions in rats. Brain Res..

[77] Imperato A., Obinu M.C., Mascia M.S., Casu M.A., Zocchi A., Cabib S., Puglisi-Allegra S. (1996). Strain-dependent effects of dopamine agonists on acetylcholine release in the hippocampus: An in vivo study in mice. Neuroscience.

[78] Martin K.J., Vyas S. (1987). Increase in acetylcholine concentrations in the brain of ‘old’ rats following treatment with pyrithioxin (Encephabol). Br. J. Pharmacol..

